# Shunt Testing In Vivo: Illustration of Partially Obstructed Ventricular Catheter by In-Growing Choroid Plexus

**DOI:** 10.7759/cureus.9287

**Published:** 2020-07-19

**Authors:** Virginia Levrini, Zofia Czosnyka, Indu Lawes, Angelos G Kolias, Richard Mannion

**Affiliations:** 1 Neurosurgery, Addenbrooke's Hospital, Cambridge University Hospitals NHS Foundation Trust, Cambridge, GBR; 2 Clinical Neurosciences, Addenbrooke's Hospital and University of Cambridge, Cambridge, GBR

**Keywords:** cerebrospinal fluid, hydrocephalus, chiari malformation, ventriculoperitoneal shunt, neurosurgery

## Abstract

Assessing shunt function in vivo presents a diagnostic challenge. Infusion studies can be a cost-effective and minimally invasive aid in the assessment of shunt function in vivo. We describe a case of a patient who after a foramen magnum decompression for type I Chiari malformation developed bilateral posterior fossa subdural hygromas and mild hydrocephalus, eventually necessitating insertion of a ventriculoperitoneal shunt. The patient returned with symptoms that were concerning for infection of the shunt. A bedside infusion study helped confirm that the ventricular catheter was partially obstructed by in-growing choroid plexus, but also that the shunt was no longer necessary. Partial blockage due to in-growing choroid plexus was confirmed during surgery to remove the shunt. We discuss the behaviour of in-growing choroid plexus and how partial obstruction can be detected with the use of an infusion study, as well as how this compares to the pattern observed in complete shunt obstruction. The benefits of using infusion studies in the assessment of shunt function are also explored.

## Introduction

Obstructed, underdraining or overdraining shunts may disturb optimal management of hydrocephalus. Proper evaluation of shunt function in vivo often presents a challenge. Different techniques are used, including microflowmetry, ultrasonography or MRI techniques [1-3]. All of them require specialised equipment and expertise for interpretation of the results. Moreover, most of these methods rely on detection of spontaneous flow of cerebrospinal fluid (CSF) through the shunt, which may not be present at the time of testing even in a patent shunt. Reliable shunt testing in vivo is important as clinical symptoms and brain imaging data may be incongruous. In the case of the shunt functioning well, it spares unnecessary revision.

Infusion tests to assess shunt function allow quick, objective assessment of shunt function with forced accelerated flow of fluid through the shunt. Two 25 G needles are inserted transcutaneously into the shunt pre-chamber using sterile preparation and careful skin cleaning [4]. Through one needle Hartmann’s fluid is infused and pressure recording is performed through the second needle. In the case of obstruction of the intraventricular catheter, three findings are specific [5]. Firstly, baseline recorded CSF pressure is without any or with very small pulsations synchronised to the heart rate. Secondly, after starting the infusion, CSF pressure increases precipitously (within 10-20 seconds) to a value slightly above the shunt opening pressure. Thirdly, if the shunt has a subcutaneous chamber siphon control device, compression of the chamber during infusion blocks flow through the shunt and recorded pressure increases to a high value immediately (usually above 50 mm Hg with a delay of only a few seconds). 

We present a case in which ventricular catheter blockage was detected with an infusion test and the finding was confirmed during revision surgery. 

## Case presentation

A 37-year-old female patient with a symptomatic type I Chiari malformation underwent foramen magnum decompression in our department. The operation was uneventful and she was discharged home two days post-operatively. 

**Figure 1:**
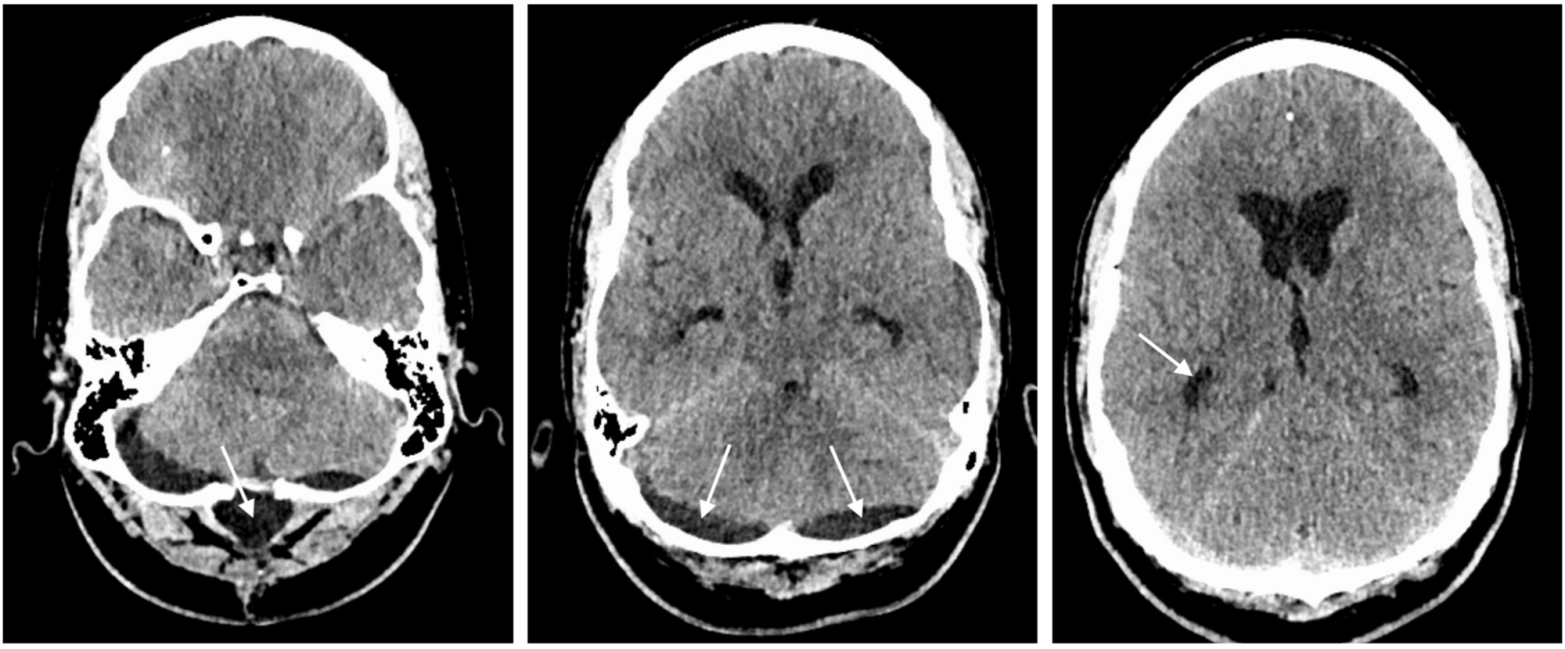
CT head two months following foramen magnum decompression. CT head demonstrating small pseudomeningocele in relation to foramen magnum decompression (arrow in left image), bilateral posterior fossa subdural hygromas (arrows in middle image) and mild hydrocephalus with dilated temporal horns (arrow in right image).

She presented two months later with a two-week history of headaches exacerbated by lying flat. A CT head showed small bilateral posterior fossa subdural hygromas and mild hydrocephalus (Figure 1). As her symptoms progressed, she underwent posterior fossa re-exploration where the craniectomy was extended with two new burr holes placed laterally on both sides, the dura was opened and the CSF collections were drained. Some of the CSF was collected and as the microscopy demonstrated CSF pleocytosis (polymorphs [PMNs] 156 x 10^6^/L, lymphocytes 24 x 10^6^/L, red blood cells 32 x 10^6^/L), she was started on IV ceftriaxone pending culture results, which later returned as no significant growth. Her headache gradually subsided over the next two days. Three days post-operatively, she developed a wound leak, which persisted despite re-suturing.

**Figure 2:**
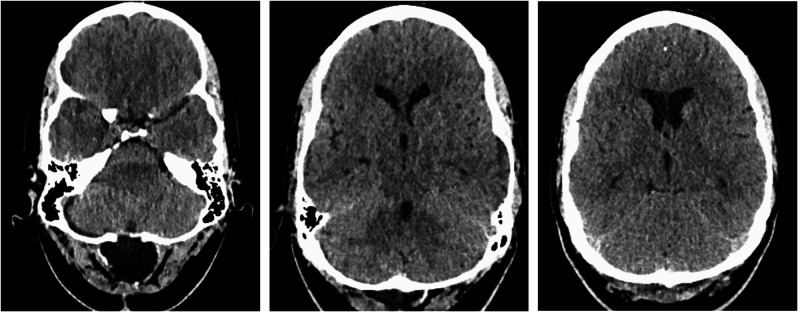
CT head after posterior fossa re-exploration surgery. Repeat CT head demonstrating almost complete resolution of the posterior fossa subdural hygromas after surgical drainage and mild hydrocephalus.

A repeat CT head showed resolution of the hygromas and an unchanged degree of mild hydrocephalus; in view of this and the persistent leak, a ventriculoperitoneal shunt (VPS) was offered to the patient (Figure 2). A right parietal VPS was inserted with a programmable valve (small Strata II valve (Medtronic, Dublin, Ireland) set at 1.5) without complications. No further wound leaks were noted and the patient was discharged home the following day. 

**Figure 3:**
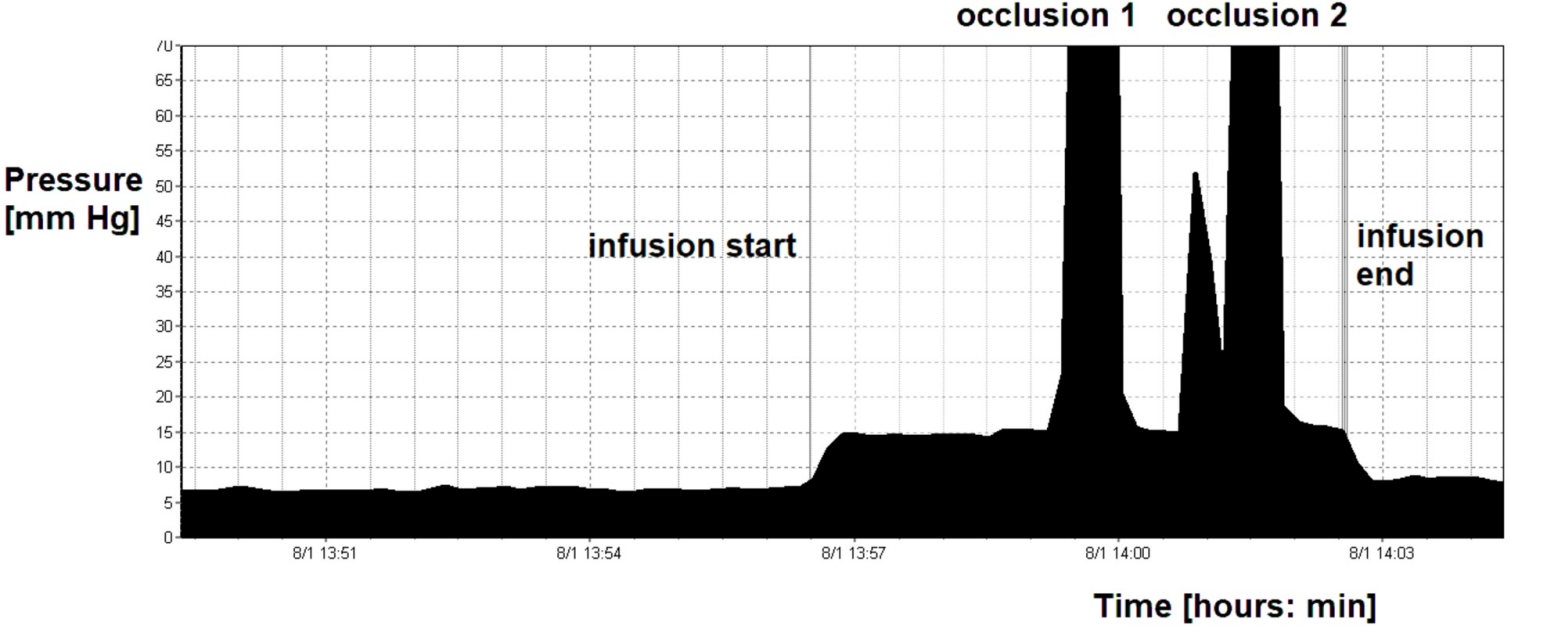
Recording of pre-chamber pressure during infusion test. During the first eight minutes, the pressure was around 7 mm Hg with minimal but detectable pulsations (around 0.1 mm Hg). During infusion, pulsations disappeared and pressure very quickly reached 15 mm Hg, which is in agreement for Strata valve working at 1.5. Further two compressions of the siphon control device were performed. During compression, pressure increased to above 150 mm Hg without delay. After end of infusion, pressure decreased to pre-infusion level and minimal pulsation re-appeared. Remarkably, after insertion of needles into the shunt pre-chamber, cerebrospinal fluid could be aspirated fluently.

The patient represented to the emergency department one month later with fever, headaches, neck pain, nausea and vomiting. Neurological examination revealed mild neck stiffness but no focal neurological signs. All incisions looked healthy. A CT head and X-rays of the shunt showed decompressed ventricles and continuity of the shunt system, respectively. The shunt pre-chamber was accessed with a 25 G butterfly needle, but no CSF could be aspirated. A small pseudomeningocele was tapped, and microscopy showed raised lymphocytes (570 x 10^6^/L) but normal PMNs (6 x 10^6^/L) and no organisms on Gram stain. Her C-reactive protein (CRP) was normal. She was started on a course of intravenous ceftazidime and vancomycin and a course of aciclovir. She was kept under close neurological observation. She continued to have fever intermittently over the next few days but remained clinically well. Hence, she was reviewed by the infectious diseases team who could not identify a source of the fever but concluded that a VPS infection could not be excluded. As the patient was initially reluctant to undergo another operation, an infusion study was organised to assess her CSF circulation. Before starting the infusion, CSF was aspirated from the shunt pre-chamber with microscopy revealing 4 x 10^6^/L PMNs and 8 x 10^6^/L lymphocytes. The infusion study results revealed partial obstruction of the ventricular catheter but normal baseline intracranial pressure (ICP), suggesting that the patient was not shunt-dependent (Figure 3).

**Figure 4:**
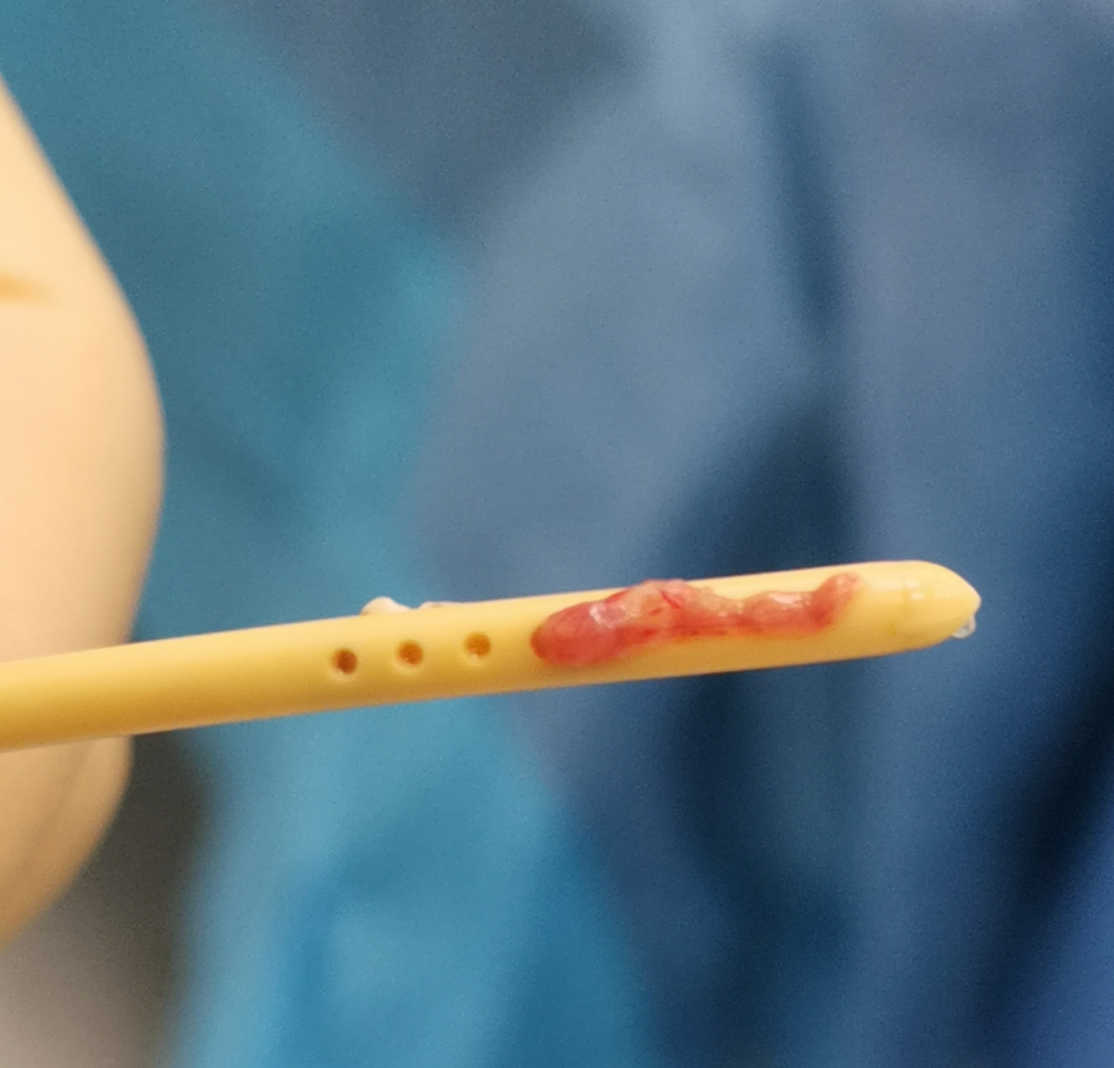
Photo of explanted ventricular catheter. After explantation of the ventricular catheter, choroid plexus can be seen in-growing through holes.

The option of removing the shunt was discussed with the patient, explaining the small possibility that she might need another VPS at a later date. The patient agreed to proceed and the shunt was removed without complications. When the ventricular catheter was removed, it was noted that choroid plexus was adherent to the ventricular catheter tip (Figure 4). The tip was cultured, with no growth found after two days incubation. The patient was well post-operatively and was discharged home after 24 hours. She remains well at four-month follow-up. 

## Discussion

**Figure 5:**
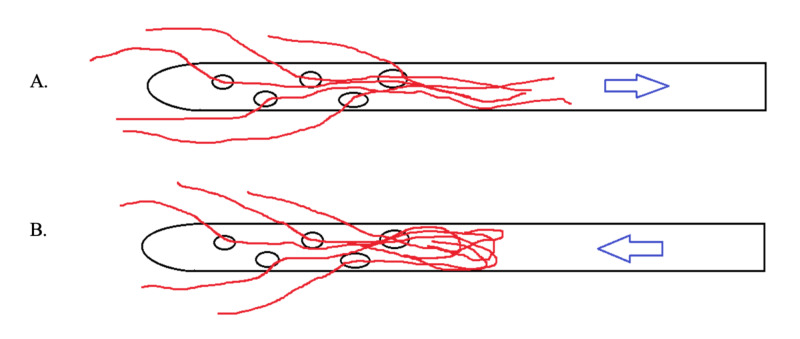
Illustration of "water weed" behaviour. (A) When choroid plexus in-grows into ventricular catheter, flow towards the shunt is partially obstructed, but possible. Aspiration of cerebrospinal fluid is possible, pulse amplitude in measured pressure is usually damped. (B) During infusion pressure in the pre-chamber increases, choroid plexus disrupts communication of pre-chamber with ventricles. Pulse amplitude in recording disappears. During occlusion of the siphon control device, shunt behaves as proximally blocked.

A number of important lessons can be learnt from this case. First, shunt blockage at the ventricular end can be detected with relative ease. In this case, the intraoperative findings confirmed the conclusion drawn from the infusion study. An in-growing choroid plexus does not necessarily completely block the shunt. Flow of CSF can be only partially blocked; therefore, aspiration of CSF from the shunt pre-chamber is possible. Choroid plexuses behave as a “water weed” in a river, aligning in a direction and not necessarily fully blocking CSF flow. Additionally, a pulse waveform at baseline can be seen; however, this is usually very small and disappears during infusion. When the pressure in the shunt pre-chamber increases during infusion, the longitudinal orientation of the in-growing plexus is disturbed and it firmly blocks the communication between the shunt and cerebral ventricles (Figure 5). The pulse amplitude disappears and the shunt starts to behave as if there is a solid ventricular block. Therefore, during occlusion, pressure in the pre-chamber rises to very high values without delay. In contrast, when the intraventricular end is completely blocked, no pulse waveform of ICP can be seen and it is impossible to aspirate any CSF from the shunt pre-chamber. Other findings are the same as in partial blockage by in-growing choroid plexus. Shunt testing in vivo is helpful in making the right decision in complex cases. In this particular case, the improperly working shunt was removed as the disturbed CSF circulation appeared to be transient based on the finding of normal baseline ICP.

The second lesson is about decision to shunt. Shunts for hydrocephalus principally facilitate poor CSF circulation. With a decision to shunt made on the basis of clinical symptoms and brain imaging, the relevant symptoms improve after shunting in approximately 60% to 90% of cases. However, in 10% to 40% of cases, the improvement is partial or absent altogether [6]. Therefore, it is logical, especially considering the low invasiveness of an infusion test, to confirm if the CSF circulation is indeed impaired before first-time shunting in equivocal cases. In the described case, such an investigation was not initially conducted because the patient had a persistent CSF leak through the recently re-opened wound, making the decision to insert a shunt straightforward. 

Finally, shunt testing in vivo can also prevent unnecessary revisions, if the shunt is found to work well during infusion test. Our recent study suggests that in approximately 50% of the tests in patients with a shunt in situ, the shunt system is patent [4]. Appropriate management will then depend on the specifics of each case but may include a change in the shunt setting, implantation of an anti-siphon device or treatment of symptoms that are due to another condition. As well as avoiding the risks associated with revision, the average financial gain by unnecessary revisions in our hospital is around one million pounds per year [4]. Infusion tests are safe. The risk of infection or other complications is less than 0.6%. 

## Conclusions

We describe a case of a partially obstructed ventricular catheter by in-growing choroid plexus. Partial blockage was ascertained with a bedside infusion study and the results confirmed during surgery. As illustrated by this patient’s case, it can be difficult to detect partial blockage using clinical picture and shunt tap only. Therefore, we discuss the expected results during infusion study in the case of partial blockage and the underlying mechanism in the case of an in-growing choroid plexus. Shunt infusion studies can assist in decision making for patients presenting with suspected shunt malfunction. 
